# Maternal high-fat diet increases the susceptibility of offspring to colorectal cancer via the activation of intestinal inflammation

**DOI:** 10.3389/fnut.2023.1191206

**Published:** 2023-05-12

**Authors:** Shimin Zheng, Jianbin Yin, Hui Yue, Lifu Li

**Affiliations:** ^1^Department of Gastroenterology and Hepatology, The Third Affiliated Hospital, Southern Medical University, Guangzhou, China; ^2^The Third School of Clinical Medicine, Southern Medical University, Guangzhou, China; ^3^Department of Orthopedics, The Third Affiliated Hospital, Southern Medical University, Guangzhou, China

**Keywords:** maternal, offspring, high-fat diet, colorectal cancer, intestinal inflammation

## Abstract

A high-fat diet plays a key role in the pathogenesis of colorectal cancer, and this effect on the gut can also occur in the offspring of mothers with a high-fat diet. In this review, we discuss the role of a high-fat diet in the pathogenesis of colorectal cancer and summarize the effects of a maternal high-fat diet on the activation of inflammation and development of colorectal cancer in offspring. Studies have found that a maternal high-fat diet primarily induces an inflammatory response in the colorectal tissue of both the mother herself and the offspring during pregnancy. This leads to the accumulation of inflammatory cells in the colorectal tissue and the release of inflammatory cytokines, which further activate the NF-κb and related inflammatory signaling pathways. Research suggests that high levels of lipids and inflammatory factors from mothers with a high-fat diet are passed to the offspring through the transplacental route, which induces colorectal inflammation, impairs the intestinal microecological structure and the intestinal barrier, and interferes with intestinal development in the offspring. This in turn activates the NF-κb and related signaling pathways, which further aggravates intestinal inflammation. This process of continuous inflammatory stimulation and repair may promote the uncontrolled proliferation of colorectal mucosal cells in the offspring, thus increasing their susceptibility to colorectal cancer.

## 1. Introduction

Colorectal cancer (CRC) is a common malignant tumor of the digestive tract. According to the global cancer statistics published by the World Health Organization (WHO) in 2020, CRC was the third most commonly diagnosed cancer (incidence: 10.0%) and the second most deadly cancer (mortality: 9.4%) (1). It is estimated that the incidence of CRC will increase by 60% by 2030, affecting ~2.2 million people (2). CRC can be divided into two main types based on the carcinogenesis process: one derived from adenomas and the other associated with inflammation (3). Notably, in both adenoma-derived CRC and inflammation-associated CRC, the continuous stimulation of inflammation and cell proliferation persists throughout the process of CRC development (4). However, the pathogenesis of CRC is not yet clear, and any external risk factors that can cause colorectal inflammation may lead to the onset and development of CRC.

Obesity is a common public health issue worldwide and is recognized as a major risk factor for cancer (5). In the United States, 5% of male cancer cases and 10% of female cancer cases among patients aged over 30 were associated with obesity from 2011 to 2015 (6). Multiple studies have found that obesity increases the risk of CRC (7–9). Obesity is mostly caused by the excessive intake of dietary fat, accompanied by impaired lipid metabolism, and often causes systemic inflammation, with the process of cell repair following inflammation being accompanied by continuous cell proliferation. This high-fat-diet-induced inflammation is suggested to be closely associated with the onset and development of CRC. Furthermore, a maternal high-fat diet has been shown to increase the incidence of malignant tumors in the offspring (Table 1). In previous studies, increased risks of breast cancer were found in the offspring of mothers who consumed a high-fat diet, and an increased risk of tumor recurrence was found in the offspring of breast cancer patients with a high-fat diet (10, 11). Studies have also shown a maternal high-fat diet to be a risk factor for pancreatic cancer and primary liver cancer (12–14) as well as colorectal inflammation and CRC (15) in offspring. This finding has great significance for in-depth investigation of the pathogenesis and treatment of CRC.

**Table 1:** Study on the relationship between high-fat diet of parents and malignant tumor in offspring.

Study	Animals	Groups	Sacrifice age	Results	Test
Zhang X, et al. Endocrine Related Cancer (2020) (11)	SD rats	G1: MNC + DMBA (F1) *n* = 23 G2: MHFD + DMBA (F1) *n* = 29	23 weeks	The total number of mammary tumors, recurred after tamoxifen (TAM) treatment: G2 > G1 (*p* < 0.05)	Chi-square test
da Cruz RS, et al. Endocrine Related Cancer (2019) (12)	P48^Cre/+^/Kras^G120/+^ mice	G1: PNC (F1) *n* = 58 G2: PHFD (F1) *n* = 52 G3: MNC (F1) *n* = 44 G4: MHFD (F1) *n* = 34	24 weeks	Ratio of high/low grade pancreatic intraepithelial neoplasia and invasive Pancreatic ductal adenocarcinoma incidence: G2 > G1 (*p* < 0.05) G4 > G3 (*p* < 0.05)	Student’s *t* test
Sun Y, et al. Journal of Hepatology (2020) (13)	C57BL/6 mice	G1: MNC + DEN (F0) *n* = 7 G2: MHFD + DEN (F0) *n* = 7 G3: MHFD + DEN (F1) *n* = 12 G4: MHFD + DEN (F2) *n* = 9	40 weeks	Quantification results of tumor: G3 > G2 > G1 (*p* < 0.05) G3 vs. G4 (*p* > 0.05)	Mann–Whitney *U* test

SD rats, Sprague–Dawley (SD) rats; DMBA, 7,12-dimethylbenz [a] anthracene; TAM, tamoxifen; DEN, diethylnitrosamine; MNC, Maternal NC; MHFD, Maternal HFD; PNC, Paternal NC, PHFD, Paternal HFD.

In this review, we used the keywords “high fat diet” or “obesity,” “offspring,” and “colorectal” to summarize the literature on the Pubmed website^1^ from 2000 to 2023. We summarize the role of a maternal high-fat diet in colorectal inflammation activation and carcinogenesis in offspring and speculate the possible mediators and signal transduction pathways involved in CRC development in offspring. This review highlights that a maternal high-fat-diet-induced CRC onset in offspring is possibly mediated by the activation of intestinal inflammation. This review explores the role of a high-fat diet in colorectal health and disease and its mechanism of action in CRC development and progression. Improving the maternal dietary structure and avoiding the long-term consumption of a high-fat diet may be necessary measures to prevent CRC in offspring.

## 2. High-fat diet promotes maternal CRC by activating intestinal inflammation

A high-fat diet has been linked to an increased incidence of CRC (16, 17). A meta-analysis of studies on CRC risk factors that followed participants for up to 10 years showed that participants with abnormally high lipid levels had a significantly increased risk of CRC compared with participants with normal lipid levels (18). Early studies have found that a high saturated fatty acid intake increases the risk of CRC or precancerous lesions and that animal fat intake is positively correlated with the risk of CRC (19, 20). Liu et al. also confirmed this finding in a retrospective cohort study of CRC patients (21). Another study showed that under the same exposure conditions to carcinogenic agents, the colorectal cells of high-fat diet-fed mice proliferated actively and the levels of the intestinal crypt cell-related proliferation factor Ki67 increased compared with the colorectal cells of normal diet-fed mice (22). In some studies, colorectal tissue exposed to a high-fat diet showed elevated expression levels of stem cell markers and a significant tendency to become cancerous (23, 24).

The susceptibility to colorectal carcinogenesis caused by a high-fat diet may be associated with inflammation caused by increased serum levels of inflammatory cytokines (25). Chronic inflammation has been shown to promote cell proliferation and repair, possibly inducing cell overproliferation and malignant transformation (26). A study found that, under the treatment of dextran sodium sulfate (DSS), the levels of the inflammatory cytokines interleukin (IL)-1β, IL-6 and tumor necrosis factor (TNF) were significantly increased in the colorectal tissue of high-fat diet mice compared with mice on the normal diet (27). Notably, the levels of colorectal inflammatory cytokines were also significantly increased in mice fed a high-fat diet without DSS compared with mice on a normal diet (28). IL-1β, IL-6 and TNF are proinflammatory cytokines secreted by macrophages. Our previous study found that mice fed a high-fat diet that were not treated with carcinogenic or pro-inflammatory drugs demonstrated visceral lipid accumulation, reduced colorectal length and significantly increased levels of macrophages and the tumor stem cell markers CD133 and CD44 in the colorectal tissue (29). This result confirmed that a high-fat diet promotes colorectal inflammation and potentially induces CRC development.

According to the above reports, intestinal inflammation is an important step in the CRC induction mechanism of a high-fat diet. Macrophages, as important immune cells, secrete the inflammatory cytokines IL-1β, IL-6 and TNF, which are involved in the NF-κb pathway, an important signaling pathway involved in the pathogenesis of CRC (30). In addition, a high-fat diet often causes intestinal dysbacteriosis, which damages the intestinal mucosal barrier. The resulting inflammatory response upregulates the levels of circulating lipopolysaccharide and TNF-α, both of which further activate the NF-κb pathway (31). The NF-κb pathway regulates inflammatory responses, and the activation of this signaling pathway may be a mechanism by which high-fat diet-related inflammatory responses promote the onset and development of CRC. Thus, the inflammation caused by a high-fat diet activates the relevant signaling pathways and further destroys the intestinal barrier. The process of continuous proliferation and repair in the intestinal mucosa may cause the uncontrolled proliferation of colorectal mucosal cells, which may induce the proliferation of tumor stem cells, eventually leading to CRC. You may insert up to 5 heading levels into your manuscript as can be seen in “Styles” tab of this template. These formatting styles are meant as a guide, as long as the heading levels are clear, Frontiers style will be applied during typesetting.

## 3. Maternal high-fat diet causes lipid metabolism disorder in offspring

A high-fat diet not only increases blood lipid levels and causes visceral fat accumulation in parents but also affects lipid metabolism in offspring during pregnancy. Epidemiological studies have found that maternal obesity and weight gain during pregnancy increase the risk of adverse outcomes, such as obesity, in infants (32–35), and children with obesity are more likely to have lifelong obesity, which compromises their health and life expectancy (36). Obesity and metabolic abnormalities have a genetic predisposition, and most studies have confirmed that babies born to mothers who consumed a high-fat diet during pregnancy were more likely to develop metabolic syndrome and exhibit weight gain and elevated lipid levels that may continue into adulthood (36–39). Similar findings have been found in animal studies wherein the offspring of mice fed a high-fat diet were more likely to develop neonatal obesity than the offspring of mice fed a normal diet. When mice were fed a high-fat diet during pregnancy, their offspring showed significant metabolic abnormalities such as visceral fat accumulation and elevated blood lipids, even after the offspring were fed a normal diet. Similar effects were also observed in their grandpups under normal diet. Preperitoneal fat deposition and systemic inflammation were also found in the offspring mice (25). Other studies have found that a maternal high-fat diet is a risk factor for systemic inflammation and hepatic steatosis in offspring mice (40). Wang et al. found that these offspring mice had not only impaired lipid metabolism but also fatty degeneration of the liver and intestinal flora disorder (41). All of the above studies indicate that a maternal high-fat diet has a significant effect on lipid metabolism in the offspring, which can lead to elevated lipid levels, fat accumulation in multiple organs and systemic inflammation.

The intestinal flora also plays an important role in lipid metabolism disorder. A high-fat diet strongly affects the intestinal flora, and the intestinal flora further has a feedback effect on the process of lipid metabolism. Intestinal microbes can regulate the function of fat cells and affect the nutritional intake and metabolism of the host to regulate the energy balance in the body (42). A high-fat diet often causes intestinal dysbacteriosis (43, 44), which can in turn disrupt the host energy balance by dysregulating fat cells and further promote lipid metabolism disorders. In multiple studies, the gut microbial composition of offspring and grandpups in the maternal high-fat diet group was different from that in the control group (25, 27, 45). The changes in the intestinal flora of the offspring of mice fed a high-fat diet were mainly manifested as an increase in the abundance of *Firmicutes* (mainly *Lactococcus*), a decrease in the abundance of γ-*Proteobacteria* (mainly *Escherichia*) and a partial increase in the abundance of *Betaproteobacteria* (mainly *Comamonas*), which are associated with a high-fat environment in the intestine (46). Furthermore, the offspring of mice treated with probiotics showed improved composition of the intestinal flora, improved intestinal microecological function and significantly decreased body weight and lipid levels compared with the offspring of mice fed a high-fat diet (47). It can be concluded that the changes in the intestinal flora of offspring caused by a maternal high-fat diet play an important role in promoting lipid metabolism disorder in the offspring.

The exact mechanism of lipid metabolism disorder in offspring caused by a maternal high-fat diet is not clear. Research indicates that some of the energy in the pregnant woman is converted to and stored as fat in the fetus. Mothers with pre-existing metabolic disorders or metabolic disorders developed during pregnancy have elevated blood lipid levels. This metabolic imbalance leads to excessive energy storage in the fetus during pregnancy. The insulin resistance caused by metabolic imbalance during pregnancy further elevates maternal blood glucose and blood lipid levels, resulting in excessive transplacental transfer of glucose, triglycerides and fatty acids to the fetus (48). The placenta is the key organ for nutrition and waste exchange between mother and fetus, and thus, maternal nutrition and placental status play a crucial role in regulating the growth and development of the fetus (49, 50). Fatty acids in the maternal circulation can pass to the fetal circulation through transplacental transfer (51). Zhu et al. found that the fetuses of obese ewes had significantly increased levels of circulating free fatty acids, cholesterol and triglycerides compared with the fetuses of normal ewes (52). Impaired maternal lipid metabolism may affect fetal energy metabolism through the transplacental route and cause lipid metabolism disorders in offspring. In addition, maternal obesity causes increased levels of glucose and amino acid transporter transcription in the placenta as a result of impaired placental function (53, 54). Impaired placental function may also cause metabolic abnormalities in offspring. A maternal high-fat diet indices a series of inflammatory reactions, such as oxidative stress, in the placenta (55). Macrophages activated in the placenta dysregulate the blood vessels of the placenta, resulting in decreased vascular maturity and decreased blood flow in the placenta (52, 53); this further aggravates hypoxia and inflammatory reactions in the placenta, leading to maladaptation of the placenta and impaired placental function (31). A study that isolated umbilical cord-derived mesenchymal stem cells found that infants with inherited obesity had impaired fatty acid metabolism in the stem cells (56), which may be a manifestation of impaired placental function.

The above studies suggest that a maternal high-fat diet disrupts lipid metabolism in the offspring via the transplacental transport of high levels of lipid into fetal circulation. In addition, metabolic disorders in the offspring may be related to placental function. The disrupted maternal lipid metabolism activates inflammation in the placenta, which damages placental function and in turn impairs offspring lipid metabolism. In addition, intestinal dysbacteriosis caused by maternal lipid metabolism disorder is also reflected in the offspring intestinal microecology, which further affects lipid metabolism in the offspring. However, the specific mechanism of lipid metabolism disorder in offspring caused by a maternal high-fat diet still needs to be clarified.

## 4. Maternal high-fat diet causes intestinal inflammation and damages the epithelial barrier function of the intestinal mucosa in offspring

A maternal high-fat diet can disrupt lipid metabolism in offspring, increasing their susceptibility to intestinal inflammation, and has been shown to promote the early onset of intestinal inflammation in offspring (57). For example, Xie et al. demonstrated that the offspring of mice fed a high-fat diet were more susceptible to DSS-induced colitis in adulthood than those of mice fed a normal diet despite being fed a normal diet since birth (27). Furthermore, the gut of these offspring of mice fed a high-fat diet showed severe intestinal ulcers, together with increased mRNA expression of the pro-inflammatory cytokines TNF-α, interferon (IFN)-γ, IL-1β, IL-12, IL-6, IL-17 and IL-22 and decreased mRNA expression of the anti-inflammatory cytokine IL-10. Compared with the offspring of mice fed a normal diet, these offspring had greater weight loss, a higher disease activity index, a significantly shorter colorectum, greater intestinal permeability and inflammation and a significantly lower survival rate (58, 59). In sheep, in addition to elevated levels of proinflammatory cytokines and chemokines in the circulation of parents fed a high-fat diet, the colorectal tissue of their fetuses showed increased expression levels of genes encoding inflammatory signaling proteins and, consequently, increased intestinal permeability after birth. These offspring also had decreased expression levels of intestinal compact junction protein, decreased goblet cell density and a decreased ileum villi–crypt ratio (60). Xue et al. studied the 16-week-old offspring of mice fed a high-fat diet and found that, compared with mice on the normal diet, the serum and intestinal levels of inflammatory factors were significantly increased, while the level of tight junction protein in the intestinal barrier epithelium was decreased. Morphologically, the number of cup cells in the ileum and the height of the intestinal epithelial villi were decreased, while the depth of the intestinal crypt was increased. The decreased villus–crypt ratio suggested intestinal mucosal injury (61). Xie et al. studied the 3-week-old offspring of high-fat diet-fed mice and found that the expression of tight junction protein in the intestinal tissue was inhibited and the membrane localization of the protein was also changed. In addition, the IgA expression cells and SIgA levels in the gut were significantly decreased, while the levels of the inflammatory cytokines IL-6 and TNF-α were significantly increased. (27) This series of studies confirmed that a maternal high-fat diet induces intestinal inflammation and damages the intestinal mucosal barrier in offspring.

A maternal high-fat diet may promote the progression of intestinal inflammation and induce intestinal carcinogenesis in the offspring through vertically transmitted disturbance of the intestinal flora. The establishment of the intestinal flora begins in the fetus. Studies have found that the intestinal microecological state can be passed directly from mother to offspring, and this intestinal colonization often occurs during fetal intrauterine development, delivery and lactation (62, 63). The transfer of intestinal flora disturbances from parents fed a high-fat diet to their offspring may be related to inherited high lipid levels in the offspring (64, 65). Gohir et al. found that abnormal intestinal development in the offspring of parents fed a high-fat diet can also induce intestinal microecological disorder (31). These findings are consistent with the phenomenon of “hereditary intestinal flora disorder” proposed by Barbosa et al., which may also be one of the reasons for the increased susceptibility to intestinal inflammation observed among the offspring of parents fed a high-fat diet. As an integral part of the intestinal environment, the intestinal flora is closely involved in the regulation of the intestinal barrier and lipid metabolism. A high-fat diet causes intestinal dysbacteriosis, which in turn promotes colorectal inflammation and even carcinogenesis (66, 67). These offspring exhibit a damaged intestinal barrier caused by the metabolites of disordered intestinal flora, which also increases susceptibility to intestinal inflammation and carcinogenesis.

## 5. Maternal high-fat diet increases the susceptibility of offspring to CRC

The offspring of mothers fed a high-fat diet show an increased risk of cancer such as breast cancer, pancreatic cancer and CRC compared with the offspring of mothers fed a normal diet (10–14). A large prospective cohort study from the United States found that maternal obesity and gestational weight gain are important risk factors for CRC in offspring (15). Many animal experiments have found that a maternal high-fat diet causes visceral fat accumulation in offspring (68, 69). This visceral fat accumulation, in turn, significantly increases the risks of CRC and cancer-related death (70). The specific mechanism by which a maternal high-fat diet causes CRC in offspring remains unclear, but the abovementioned studies confirm that a maternal high-fat diet can cause lipid metabolism disorders and intestinal inflammation in offspring. Thus, the increased susceptibility to intestinal cancer in offspring may be attributable to impaired lipid metabolism.

The intestinal growth and development of the fetus begins during pregnancy. A maternal high-fat diet may affect the intestinal growth and development of offspring through inflammation. In rats, it was found that a maternal high-fat diet not only increased the levels of intestinal inflammatory factors but also significantly changed the intestinal tissue morphology in the offspring (71). Studies have shown that placental oxidative stress caused by a high-fat diet not only damages placental blood vessels and induces placental hypoxia but also impairs placental function, thus affecting the intestinal growth and development of the fetus (31). Xie et al. found that the villus length and crypt depth of 3-week-old offspring of mice fed a high-fat diet were significantly smaller than those of mice fed a normal diet (27). Intestinal pluripotent stem cells present in the intestinal crypts can differentiate into various types of mature intestinal cells. The shallow crypt depth of 3-week-old mice might be due to restricted intestinal development in the fetal stage. However, lactation promotes the proliferation of intestinal stem cells, which further differentiate rapidly under the stress of weaning, eventually showing increased intestinal crypt depth and decreased intestinal villus height. (72) Xue et al. found that the height of intestinal epithelial villi was decreased and the depth of intestinal crypts was increased in 16-week-old offspring of mice fed a high-fat diet (27). These findings suggest that, without the influence of maternal factors such as the placenta and lactation, the intestinal growth and development of offspring after weaning are still continuously regulated. The process of cell proliferation and differentiation is accelerated due to a high-fat diet, which increases the proportion of immature intestinal epithelial cells and goblet cells, thus increasing intestinal permeability and resulting in impaired intestinal barrier function. The above studies indicate that a maternal high-fat diet not only affects the intestinal growth and development of the fetus during pregnancy, but also has a continuous effect on the intestinal growth and development of the offspring even after birth and weaning, which may be a manifestation of persistent intestinal inflammation. This developmental abnormality is manifested by accelerated cell proliferation and differentiation, which may increase the susceptibility of offspring to intestinal malignancies.

The molecular mechanism of action of a maternal high-fat diet on the offspring’s gut is not completely clear. Studies have demonstrated that a maternal high-fat diet activates the NF-κb pathway in the offspring’s nervous system, leading to nervous system inflammation (73). Similar, NF-κb pathway activation has also been found in the gut of the offspring of parents fed a high-fat diet, which is often accompanied by significant intestinal inflammation (31). As a classic inflammatory pathway, the NF-κb pathway is closely involved in the inflammatory response caused by impaired lipid metabolism. Thus, NF-κb pathway activation has been suggested as a mechanism by which high-fat-diet-induced inflammation promotes CRC development. Inflammatory factors in the maternal circulation and in the placenta can be transmitted through the placenta to the fetal circulation (51, 74), where these may activate the NF-κb pathway in the offspring (59). In addition, lipid metabolism disorder is a known risk factor for CRC. The high level of lipid and impaired lipid metabolism in the offspring of parents fed a high-fat diet may also increase the risk of CRC via colorectal inflammation. It was found that the increase in free fatty acid concentration in the fetal circulation can enhance the NF-κb signal. (52, 75) The resulting NF-κb pathway activation may further promote colorectal inflammation and carcinogenesis in the offspring.

Taken together, these studies suggest that carcinogenesis in the colorectal tissue of the offspring of parents fed a high-fat diet may be due to mucosal barrier destruction, intestinal dysbacteriosis, and abnormal intestinal growth and development caused by high-fat-diet-induced intestinal inflammation. The activation of the NF-κb pathway may play an important role in this process. This persistent and progressive inflammatory response may be an important factor in the increased susceptibility to CRC in the offspring of parents fed a high-fat diet (Table 2).

**Table 2:** Study on offspring intestinal lesions caused by high-fat diet of parents.

Study	Animals	Group	Sacrifice age	Results	Pathway
Xie R, et al. Frontiers in Immunology (2018) (27)	C57BL/6 mice	G1: PNC + DSS (F1) *n* = 6 G2: PHFD + DSS (F1) *n* = 6	3 weeks	Inflammation of colon, damage of intestinal barrier, dysplasia of colon, dysbacteriosis G1 > G2 (*p* < 0.05)	
		G3: PNC + DSS (F1) *n* = 8 G4: PHFD + DSS (F1) *n* = 11	8 weeks	Colotis DAI score, inflammation of colon G4 > G3 (*p* < 0.05)	
Gruber L, et al. Inflammatory Bowel Diseases (2015) (57)	C57BL/6 N mice & TNFΔARE/WT Mice^**^	G1: MNC (F1) *n* = 5 G2: MHFD (F1) *n* = 5	8 weeks	Histopathological scores of distal ileum, inflammation of terminal ileum G2 > G1 (*p* < 0.05)	
Bibi S, et al. Obesity (Silver Spring, MD) (2017) (59)	C57BL/6 mice	G1: PNC + OHFD + DSS (F1) *n* = 9 G2: PHFD + OHFD + DSS (F1) *n* = 9	15 weeks	Colotis DAI score, histopathological scores of colon, inflammation of colon G2 > G1 (*p* < 0.05)	NF-κb activation in colon
Yan X, et al. Inflammatory Bowel Diseases (2011) (60)	Sheep	G1: PNC (F1) *n* = 6 G2: PHFD (F1) *n* = 6	22.5 ± 0.5 months	Inflammation of colon G2 > G1 (*p* < 0.05)	NF-κb activation in colon
Xue Y, et al. The Journal of Nutritional Biochemistry (2014) (61)	NOD/ShiLtJ mice	G1: PNC (F1) *n* = 8 G2: PHFD (F1) *n* = 8	8 weeks	Histopathological scores of ileum G2 > G1 (*p* < 0.05)	
		G1: PNC (F1) *n* = 8 G2: PHFD (F1) *n* = 8	16 weeks	Impaired intestinal barrier function G2 > G1 (*p* < 0.05)	
Gohir W, et al. The Journal of Physiology (2019) (31)	C57BL/6 mice	G1: PNC (F1) *n* = 14 G2: PHFD (F1) *n* = 14	Placenta and fetus	Impaired placenta function, dysplasia of placenta: G2 > G1 (*p* < 0.05)	NF-κb activation in fetal intestines

DSS, dextran sodium sulfate; PNC, paternal NC; PHFD, paternal HFD; MNC, maternal NC; MHFD, maternal HFD; OHFD, offspring HFD. C57BL/6 N mice & TNFΔARE/WT mice^**^: C57BL/6 N mice and TNFΔARE/WT mice mate to give birth to F1.

## 6. Conclusion

**Figure 1:**
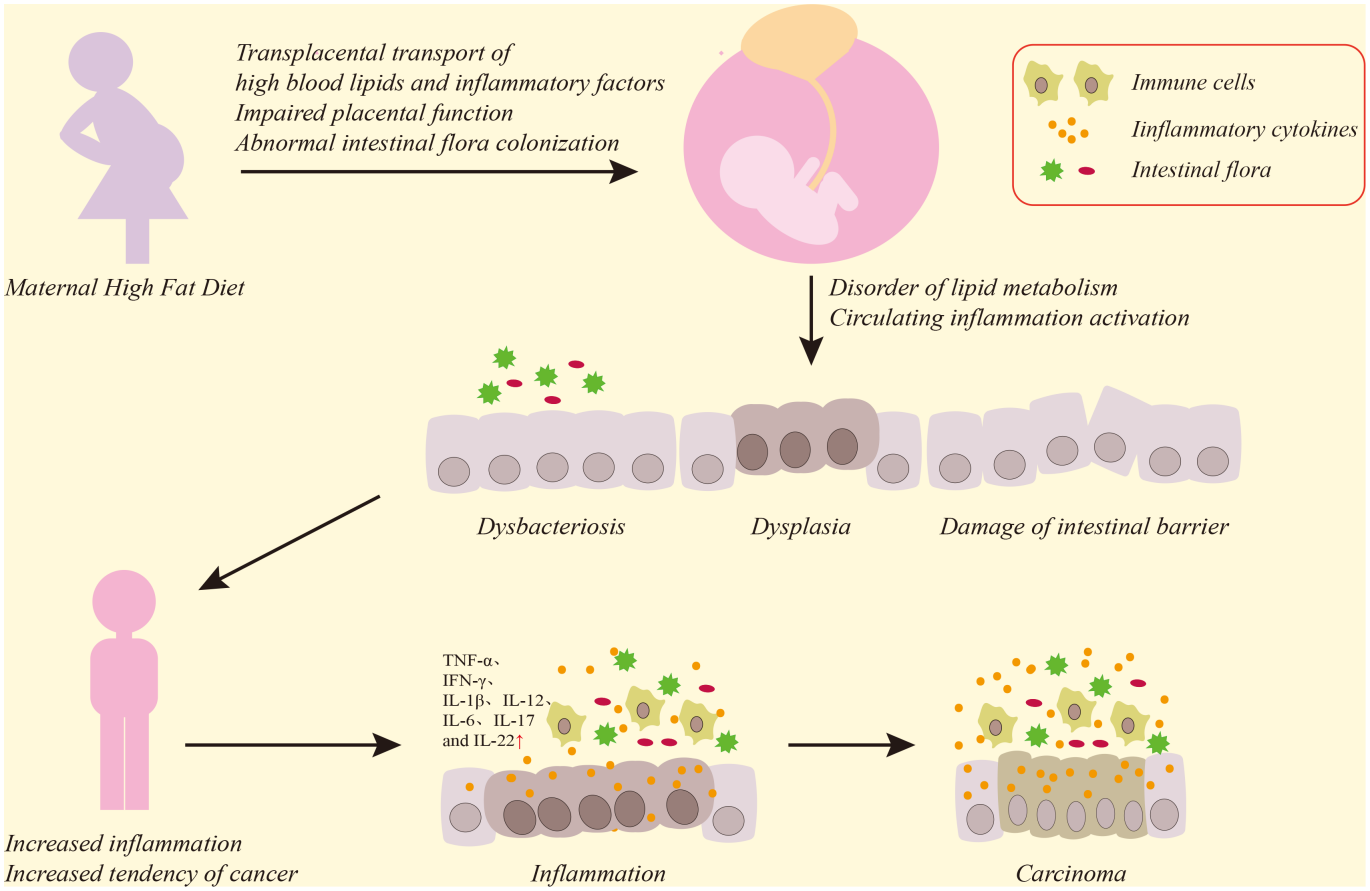
Diagram of the effect of a maternal high-fat diet on the offspring CRC. Maternal high-fat diet causes high levels of serum lipids and inflammatory factors to transfer through the placenta to offspring, damaging placental function, and phenomena such as maternal intestinal flora disorder will also be transmitted to offspring, which will lead to dysbacteriosis and functional damage to the colorectal mucosal barrier in the offspring’s colon, affect the normal growth and development of the colon, and induce intestinal inflammatory reactions. Persistent destruction of colorectal inflammation and uncontrolled proliferation of mucosal cells will ultimately lead to CRC.

This review discusses the association of a high-fat diet with CRC development and summarizes the role of a maternal high-fat diet in colorectal inflammation and CRC development in offspring. Our review reveals that a high-fat diet increases visceral fat accumulation in the host, which induces inflammatory cell aggregation and inflammatory cytokine release. This further activates the NF-κb and related signaling pathways, thereby increasing susceptibility to CRC. A maternal high-fat diet also elevates blood lipids and visceral fat accumulation in the offspring via the transplacental transfer of high levels of lipid from the maternal serum, and may impair placental function, causing metabolic disorders in the offspring. These changes also lead to intestinal dysbacteriosis in the offspring, which further aggravates lipid metabolism disorder. The impaired lipid metabolism and maternal and placental inflammatory factors in offspring circulation cause colorectal and systemic inflammation in the offspring. Colorectal inflammation impairs the intestinal barrier, hinders the normal growth and development of the intestine, accelerates the proliferation and differentiation of intestinal pluripotent stem cells and further aggravates intestinal dysbacteriosis. This process of continuous inflammatory stimulation and repair leads to the activation of the NF-κb signaling pathway, the uncontrolled proliferation of colorectal mucosal cells and, eventually, CRC onset (Figure 1).

At present, the age of diagnosis of CRC in the world is gradually getting younger, and the therapeutic effect of CRC is not ideal. This may be closely related to the diverse pathogenesis of CRC. Few studies have focused on the effect of maternal high-fat diet on offspring colorectal. A series of changes caused by high fat diet in parents may increase colorectal carcinogenesis in offspring, but the related mechanism is not clear at present. Further research to understand the cellular and molecular mechanisms of action of a high-fat diet in CRC development and the role of a maternal high-fat diet in colorectal inflammation and increased CRC susceptibility in offspring is warranted. These studies contribute to the discovery of potential therapeutic targets for CRC and the development of new therapeutic strategies for CRC based on these targets, which may be helpful to improve the efficacy of CRC. Such studies could contribute to the discovery and development of new therapeutic strategies for CRC.

## Author contributions

SZ, HY, and LL contributed to conception and design of the study. SZ and JY collect and organize the literature. SZ wrote the first draft of the manuscript. JY, HY, and LL wrote sections of the manuscript. All authors contributed to the article and approved the submitted version.

## Conflict of interest

The authors declare that the research was conducted in the absence of any commercial or financial relationships that could be construed as a potential conflict of interest.

## Publisher’s note

All claims expressed in this article are solely those of the authors and do not necessarily represent those of their affiliated organizations, or those of the publisher, the editors and the reviewers. Any product that may be evaluated in this article, or claim that may be made by its manufacturer, is not guaranteed or endorsed by the publisher.

## Footnotes

^1^ https://pubmed.ncbi.nlm.nih.gov/
